# A Note on Some Health-Related Outcomes in Small Ruminant Farms with Common Grazing with Wildlife Ruminants

**DOI:** 10.3390/ani15243579

**Published:** 2025-12-12

**Authors:** Eleni I. Katsarou, Charalambia K. Michael, Konstantinos V. Arsenopoulos, Dafni T. Lianou, Dimitra V. Liagka, Vasia S. Mavrogianni, Elias Papadopoulos, George C. Fthenakis

**Affiliations:** 1 Veterinary Faculty, University of Thessaly, 43100 Karditsa, Greece; elekatsarou@vet.uth.gr (E.I.K.); dlianou@vet.uth.gr (D.T.L.); vmavrog@vet.uth.gr (V.S.M.); 2 School of Veterinary Medicine, European University of Cyprus, Engomi, Nicosia 2404, Cyprus; cha.michael@euc.ac.cy; 3 Laboratory of Parasitology and Parasitic Diseases, School of Veterinary Medicine, Faculty of Health Sciences, Aristotle University of Thessaloniki, 54124 Thessaloniki, Greece; arsenopoulos.k@unic.ac.cy (K.V.A.); eliaspap@vet.auth.gr (E.P.); 4 Faculty of Animal Science, University of Thessaly, 41110 Larissa, Greece; dliagka@uth.gr

**Keywords:** abortion, diarrhoea, epg counts, goat, sheep, wildlife

## Abstract

The specific objective of the current paper was the description of some health-related outcomes in sheep and goat farms in Greece with common grazing with wildlife ruminants. Faecal samples were collected and information regarding health management applied on farms was obtained from sheep and goat farms located throughout Greece (325 and 119 farms, respectively). Common grazing of livestock (sheep, goats) with wildlife ruminants (roe deer, red deer) was reported in 9.2% of farms. Faecal epg counts in farms with common grazing with wildlife ruminants were higher than in farms with no common grazing: respective median values were 270 epg versus 150 epg; also, there was a tendency for faecal counts >300 epg to be recorded more frequently among the former farms (36.1% versus 23.1% of farms). Median annual incidence of cases of abortion and of cases of diarrhoea in lambs/kids was significantly higher in farms with common grazing with wildlife ruminants: 1.7% and 9.0%, respectively, versus 0.0% and 1.7%, respectively, in farms with no common grazing. The findings have revealed associations of common grazing with wildlife ruminants with the health of sheep and goats. These can have implications in the health management of farms, for example, in the administration of anthelmintic treatments and in the development of vaccination programmes in livestock farms.

## Simple Summary

The specific objective of the current paper was the description of some health-related outcomes in sheep and goat farms in Greece with common grazing with wildlife ruminants. The findings have revealed associations of common grazing with wildlife ruminants with the health of sheep and goats. Specifically, parasitic burdens, incidence of cases of abortion, and incidence of cases of newborn diarrhoea were found to be significantly higher in farms with common grazing with wildlife ruminants (roe deer, red deer). The findings have implications in the health management of farms, for example, in the administration of anthelmintic treatments and in the development of vaccination programmes in livestock farms.

## 1. Introduction

Small ruminant farming is the largest animal production business in the primary sector in Greece and contributes 18% of the total income of the sector and with 0.8% of the country’s gross domestic product [1]. This is the result of the significant numbers of sheep and goats in the country, which account for around 6.5% and 22.0%, respectively, of the total number of small ruminants in Europe [2,3].

Significant interactions can take place between small ruminants and wildlife, as 88% of sheep and goat farms in Greece are farmed under non-intensive management systems [4], ([5], Table S1), which thus involve grazing of livestock for varying periods daily. During that time, sheep and goats may share the same lands and pastures with wildlife mammals, which can lead to potential transmission of pathogens between livestock and wildlife mammals.

Apart from the potential transmission of gastrointestinal pathogens during grazing, there are various other interactions that can occur between sheep/goats and wildlife animals in relation to transmission of pathogens. The predation on grazing small ruminants by wildlife canids can lead to completion of the life cycle of various parasitic species, for example, *Echinococcus granulosus* or *Taenia multiceps*. Moreover, various arthropod species can infest grazing sheep or goats, thus disseminating pathogens from wildlife ruminants, for example, *Babesia* spp. through infestation by *Ixodes ricinus*.

Despite the above, there has been little relevant work in Greece. An early study discussed the potential overlap in feeding preferences between hares and sheep/goats in shrubland areas of Greece [6]. Billinis [7] reviewed the potential dissemination of microbial pathogens between wildlife and small ruminants, and Vasileiou et al. [8] discussed the transmission of parasites between those species. Further, Chatzopoulos et al. [9] reported cases of bluetongue in wild cervids in Greece and discussed the findings within the frame of the extensive outbreak of the infection that occurred in small ruminants in the country during 2014–2015. More recently, Petridou et al. [10] studied the feeding habits of wolves in central Greece and reported that goats and sheep were the mainly predated livestock species by these wild mammals (for use as feed); more recently, the same authors studied prophylactic measures to reduce livestock predation in sheep and goat farms [11].

The specific objective of the current paper was the description of some health-related outcomes in sheep and goat farms in Greece with common grazing with wildlife ruminants. A systematic and wide evaluation of potential associations of the practice with health problems in sheep and goats in Greece has not been reported thus far.

## 2. Materials and Methods

A cross-sectional study was performed across all 13 administrative regions of continental and insular Greece. Visits by the investigators were made to 444 small ruminant farms (325 sheep flocks and 119 goat herds) (Figure S1). The flocks and herds were included in the study on a convenience basis, specifically, the willingness of farmers to participate in the study and receive a visit for obtaining information and for sample collection [5]. Information was obtained by means of an interview with the farmer, which was performed using a structured detailed questionnaire (Tables S2 and S3) [5]; specifically, data and information were collected regarding management practices applied and health problems prevalent on the farms [5]. All the visits were carried out in association with veterinarians who supervised the farms and had arranged these visits with the farmers; these veterinarians accompanied the researchers during the visits and also verified information obtained from the farmers during the interview [5,12]. Visits to farms were made during all four seasons of the year, specifically during autumn (*n* = 41), winter (*n* = 111), spring (*n* = 151), and summer (*n* = 141).

Moreover, during the visit, faecal samples were collected from adult ewes and does on the farms [5] and were transported from the farm to the laboratory under storage into portable refrigerators. The following parasitological tests, which started within 48 h after the collection of samples, were performed in these samples: McMaster technique, flotation method, sedimentation technique, and coproculture; standard parasitological techniques were applied [13,14], as detailed before [5]. Each of the first four tests were applied in quadruplicates, whilst coproculture was performed once. Faecal epg (eggs per gram) counts, which provide an indirect measure to estimate the ‘worm burden’ for gastrointestinal nematode parasites in sheep and goats, were obtained. Subsequently, a binary outcome variable of ‘>300 epg’ versus ‘≤300 epg’ was also calculated based on the epg values obtained. Also, the presence of various other internal parasites (*Dicrocoelium dendriticum*, *Fasciola hepatica*, *Paramphistomum cervi*, *Moniezia* spp., *Teladorsagia* spp., *Haemonchus contortus*, *Trichostrongylus* spp., *Chabertia* spp., *Cooperia* spp., *Bunostomum* spp., *Nematodirus* spp., *Strongyloides papillosus*, *Trichuris* spp. and lungworms) was evaluated at the farm level. Finally, the proportion of the various species of the Trichostrongylidae family helminths in the faecal samples was also assessed.

The annual incidence rate for cases of abortion (among ewes/does), cases of lameness (among adult animals), and cases of diarrhoea (among lambs/kids born on the farms and to up to the age of slaughter (in Greece, 60 to 65 days for lambs and 75 to 90 days for kids)) were calculated. This was performed based on information obtained from the answers of the farmers during the interview and the relevant details collected from the veterinarians, who were supervising the farms and were accompanying the researchers during the respective visits [12]. During assessment, both the presence of cases of each of these three problems and their annual incidence rate were considered.

Data were entered into Microsoft Excel and analysed using SPSS v. 27 (IBM Analytics, Armonk, NY, USA). Initially, basic descriptive analysis was performed. Then, the significance of the association of the variable ‘common grazing of livestock (sheep/goats) with wildlife ruminants’ for various health-related target variables in the farms was assessed in univariable analyses. Comparisons between frequencies were performed by using Pearson’s chi-square test, comparisons between continuous data were performed by using the Mann–Whitney or the Kruskal–Wallis tests, and correlations were assessed by using Spearman’s rank correlation. For the analysis of results of parasitological examinations, only farms in which anthelmintics had not been administered during the two months prior to sampling were taken into account. Climatic variables prevailing at the location of each farm were derived from ‘The POWER (Prediction of Worldwide Energy Resources) Project’ (NASA Langley Research Center (LaRC), Hampton, VA, USA) as described before [12].

When a significant association of the above variable (‘common grazing of livestock (sheep/goats) on farms with wildlife ruminants’) with health-related target variables emerged, multivariable models were applied. These were constructed by offering therein initially the above variable (‘common grazing of livestock (sheep/goats) on farms with wildlife ruminants’), as well as other independent variables related to the health management and the climatological conditions on the farms. The additional variables in the multivariable models were sourced from previously performed analyses, during which they were found to be significant predictors for the health-related outcomes under evaluation; in those previous analyses, only management or climatological variables were used, without applying to the models the variable currently under assessment (i.e., ‘common grazing of livestock (sheep/goats) on farms with wildlife ruminants’) [5,12]. The details of the multivariable models constructed (variables offered therein, variables required in the final models, control to case ratio, case-to-variable ratio) are described in Table S4. Regression analysis was applied with progressive elimination of variables found with *p* > 0.20, until no variable could be removed from the model [15] (Table S4).

In all analyses, statistical significance was defined at *p* < 0.05.

## 3. Results

### 3.1. Descriptive Findings

Common grazing of livestock (sheep, goats) with wildlife ruminants was reported in 41 farms (9.2% (95% confidence intervals (CI): 6.7–12.3%)). Wildlife ruminant species in farms with common grazing included roe deer in 36 farms (87.8%) and red deer in 16 farms (39.0%).

There was no difference between sheep or goat farms in the respective proportions of common grazing with wildlife ruminants: 9.2% (95% CI: 6.5–12.9%) versus 9.2% (95% CI: 5.2–15.8%) (*p* = 0.99). Also, there was no significant difference between farms managed under the intensive/semi-intensive system or the semi-extensive/extensive system in the respective proportions: 8.6% (95% CI: 5.6–13.0%) versus 9.9% (95% CI: 6.6–14.6%) (*p* = 0.62).

The total grazing area by livestock did not differ significantly between farms with or without common grazing with wildlife ruminants: 50 (interquartile range (IQR): 87) hectares versus 25 (IQR: 42) hectares (*p* = 0.17). Also, there were no differences between the two cohorts of farms in the maximum grazing distance from the farm: 1000 (IQR: 1700) m for both cohorts (*p* = 0.61), and in the duration of grazing during the year: 8 (IQR: 6) months and 8 (IQR: 7) months (*p* = 0.65). However, there was a clear difference in the altitude of farms with or with no common grazing: 303 (IQR: 461) m versus 155 (IQR: 255) m, respectively (*p* = 0.0006).

There were also significant differences in climate variables between locations of farms with or with no common grazing with wildlife ruminants. In general, at the former locations, lower temperatures were recorded (*p* < 0.035), whilst wetness-related variables (relative humidity and annual precipitation) were higher (*p* < 0.03), and wind speed was lower (*p* = 0.012) (Table S5).

### 3.2. Common Grazing with Wildlife Ruminants and Health-Related Outcomes

**Figure 1:**
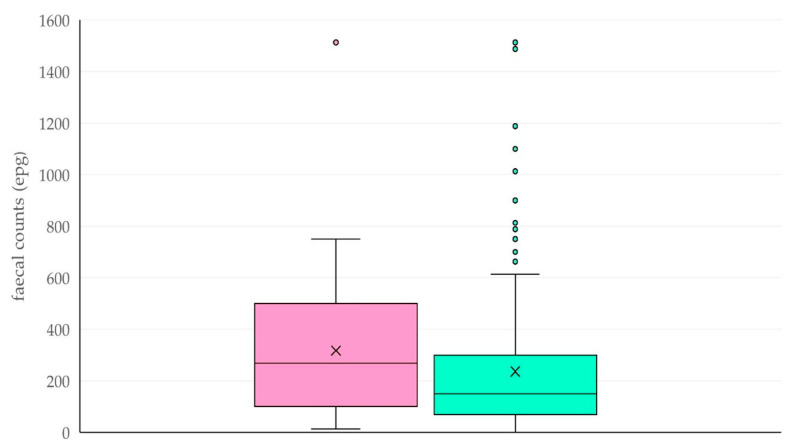
Faecal epg counts in samples obtained from small ruminants in farms where common grazing of livestock with wildlife ruminants occurred (pink plot) or did not occur (green plot).

Faecal epg counts in farms with common grazing with wildlife ruminants were higher than in farms with no common grazing: respective median values were 270 (IQR: 400) epg versus 150 (IQR: 225) epg (+80%) (*p* = 0.06) (Figure 1). This was seen at sheep (288) (IQR: 325) epg versus 157 (IQR: 212) epg, respectively, (+83%) and goat (250) (IQR: 412) epg versus 150 (IQR: 237) epg, respectively, (+67%). Moreover, there was a tendency for more frequent occurrence of faecal counts >300 epg among farms where common grazing was reported: 36.1% of those farms versus 23.1% of farms with no common grazing (*p* = 0.08). No differences were seen between the two cohorts of farms with regard to the specific helminths identified in faecal samples (Table 1).

The presence of cases of abortion was more frequent among farms with common grazing with wildlife ruminants (61.0% (95% CI: 45.7–74.4%) of farms) than among those with no common grazing (46.2% (95% CI: 41.3–51.0%) of farms) (*p* = 0.07). Moreover, the median annual incidence of cases of abortion was significantly higher in farms with common grazing with wildlife ruminants: 1.7% (4.3%) versus 0.0% (2.9%) in farms with no common grazing (*p* = 0.026). This was seen at sheep and goat farms (Table 2). Vaccination against *Brucella melitensis* infection was performed at all farms in accordance with the national relevant legislation, and vaccination against *Toxoplasma gondii* infection was not performed at any farm, whilst the proportion of farms in which vaccination against *Chlamydia psittaci* infection was performed, did not differ significantly between the two cohorts: 46.3% vs. 37.2%, respectively (*p* = 0.25) [5]. In this context, it noted that median annual incidence of cases of abortion in farms with common grazing was significantly higher when no vaccination against *C. psittaci* took place: 2.1% (IQR: 4.2%) versus 0.0% (IQR: 2.9%) (*p* = 0.046), but not when anti-chlamydial abortion vaccination had been applied in farms (*p* = 0.25) (Table 2).

Presence of cases of diarrhoea of lambs/kids was significantly more frequent among farms with common grazing with wildlife ruminants (85.4% (95% CI: 71.6–93.1%) of farms) than among those with no common grazing (56.6% (95% CI: 51.7–61.3%) of farms) (*p* < 0.0001). Moreover, the median annual incidence of cases of diarrhoea in lambs/kids was significantly higher among farms with common grazing with wildlife ruminants: 9.0% (IQR: 16.3%) versus 1.7% (IQR: 10.0%) in farms with no common grazing (*p* < 0.0001) (Table 3).

Finally, there was no difference in the frequency of presence of cases of lameness between farms with (39.0% (95% CI: 25.7–54.3%) of farms) or with no (35.0% (95% CI: 30.5–39.8%) of farms) common grazing with wildlife ruminants (*p* = 0.61). Also, the median annual incidence of cases of lameness between farms with or with no common grazing with wildlife ruminants did not differ: 0.0% (2.4%) for both cohorts (*p* = 0.80).

**Figure 2:**
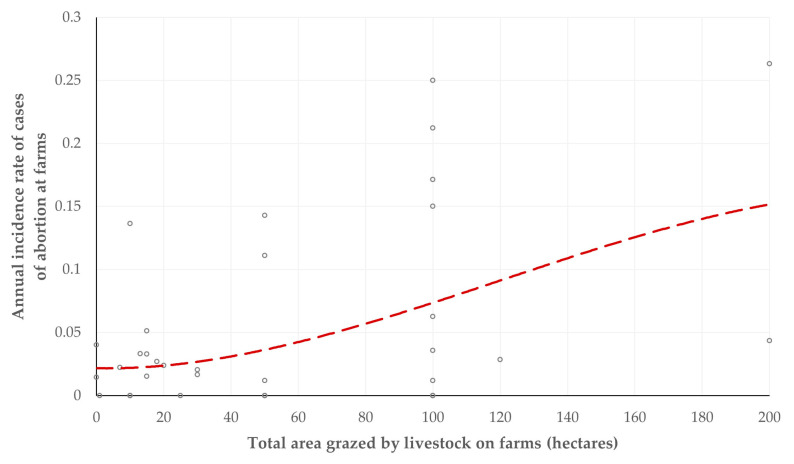
Cross-plot of the annual incidence rate of cases of abortion and the total area grazed by livestock on farms (dashed line is trendline).

Among farms in which common grazing with wildlife ruminants occurred, there was a tendency for correlation between the total area grazed by livestock and the annual incidence of cases of abortion on farms (*r_sp_* = 0.281, *p* = 0.08) (Figure 2). There were no other important (i.e., at least with a tendency) differences between the farms for the various health-related outcomes in accord with the total grazing area by livestock (*p* ≥ 0.23), the maximum distance grazed from the farm (*p* ≥ 0.35) and the duration of grazing during the year (*p* ≥ 0.11) (Table S6).

### 3.3. Significance of Common Grazing with Wildlife Ruminants as Predictor for Health-Related Variables

During the multivariable analysis for faecal counts >300 epg, the month into the lactation period at sampling (*p* = 0.003) and the application of reproductive control practices in the farm (*p* = 0.047) emerged as significant predictors (Table S7). Common grazing of livestock (sheep/goats) with wildlife ruminants was found to have only a tendency for predictor (*p* = 0.08).

During the multivariable analysis for the presence of cases of abortion, no significant predictors emerged; however, there was a tendency for the common grazing of livestock (sheep/goats) with wildlife ruminants as a predictor (*p* = 0.08). For the annual incidence of cases of abortion, the annual precipitation at farm location (*p* = 0.006) emerged as a significant predictor (Table S8); common grazing of livestock (sheep/goats) with wildlife ruminants was not significant as a predictor (*p* = 0.15).

During the multivariable analysis for the presence of cases of diarrhoea, the common grazing of livestock (sheep/goats) with wildlife ruminants (*p* = 0.001) and the annual temperature range at farm location (*p* = 0.002) emerged as significant predictors (Table S9). For the annual incidence of cases of diarrhoea in lambs/kids, the annual temperature range at farm location (*p* = 0.0004) emerged as significant predictor (Table S10); common grazing of livestock (sheep/goats) with wildlife ruminants was found to have only a tendency for a predictor (*p* = 0.07).

## 4. Discussion

### 4.1. Preamble

Despite the importance of the small ruminant industries for Greece, possible interactions between these species and wildlife present in the country have not been extensively studied, with only a few relevant papers published so far. As part of a large, countrywide investigation aiming to map the sheep and goat industries in the country, we have previously presented detailed studies on their health management and production outputs [5]. Thus far, the potential roles and associations of wildlife ruminants with the health of livestock (sheep, goats) has not been reported.

The current study looked at possible associations between the common grazing of livestock with wildlife ruminants and some health outcomes, building on previous evaluations and analyses. The novel contribution refers to the identification of the potential role of wildlife ruminants in the health problems of livestock within the ecosystem of sheep and goat farms.

The study involved an increased number of farms, which included a high number of animals (110,228 sheep and 30,192 goats [5]), with a wide geographic distribution in all the 13 administrative regions of the country. These have contributed to include a variety of management systems applied at the farms and a diversity of geographical and climatic variables, which may affect ecological considerations in relation to common grazing with wildlife mammals. The inclusion of farms on a convenience basis (i.e., the willingness of farmers to accept a visit by university personnel for an interview and collection of samples) contributed to acceptance of the visit by the farmers and to a lack of suspiciousness and distrust for the investigators, resulting in a relaxed interview. This approach allowed the inclusion of flocks and herds with farmers genuinely willing to participate in the study and to provide frank and thoughtful answers. Finally, in order to minimise possible bias, the study also used consistent methodologies, for example, interviews and collection of samples were always performed by the same investigators [5], whilst processing of faecal samples was performed by means of well-established, standardised parasitological techniques [13].

### 4.2. Faecal epg Counts

Gastrointestinal nematodes of wildlife ruminants include helminths, which infect specifically these animal species (e.g., *Ostertagia leptospicularis*, *Spiculaopteragia asymmetrica*, *Sp. spiculoptera*, *Elaphostrongylus cervi*), as well as nematodes which also infect sheep and goats, among them *Teladorsagia circumcincta*, *H. contortus*, and *Trichostrongylus axei* [14]. Therefore, there is a possibility for the occurrence of cross-infections between small ruminants and wildlife ruminants, when common grazing takes place near livestock farms. Indeed, the potential exchange of gastrointestinal nematodes between domestic and wildlife ruminants has been postulated also in a previous work, where the main nematode species identified in roe deer were those found in small ruminants (e.g., *H. contortus*), whilst wild cervid-specific species (e.g., *O. leptospicularis*) were identified with lower proportions [16].

Epg counts in faecal samples from farms where common grazing occurred were found to be higher. A possible reason for these can be the higher numbers of animals around these farms and the consequent higher stocking rates, which increase possibilities for infections of grazing sheep and goats and higher parasitic burdens [17,18]. It is also noted that in a recent study we reported that the presence of wild canids near sheep and goat farms emerged as a significant predictor for the detection of hookworms (*Uncinaria*/*Ancylostoma*) and *Toxocara canis* in farm dogs, as these parasites also infect wildlife carnivores [19].

Further, more cases with faecal samples with over 300 epg were found among farms where common grazing with wildlife ruminants occurred. The value is considered to reflect a moderate nematode parasite burden, potentially leading to decreased production, which supports the need for control measures, including administration of anthelmintic drugs [20,21].

It is noted that, for the parasitic infections, 23 comparisons were made for various helminths in the same dataset. Each comparison test has its own chance for a type I error, therefore the overall probability of making at least one false positive is higher [22,23]. Nevertheless, in the present case the comparisons performed did not produce any significant statistical associations, but only a tendency for increased faecal counts in farms with common grazing of livestock with wildlife ruminants.

All the above should be taken into account when formulating programmes for administration of anthelmintics to animals in farms where common grazing with wildlife ruminants occurs, by taking into account the grazing patterns of livestock and wildlife ruminants. In this context, possibly there may be a need to increasing the number of administrations to animals annually, modifying the timepoints of administration of anthelmintics and/or applying correctly rotation programmes of anthelmintic drugs. It is also noted that wildlife ruminants can possibly act to maintain resistant genotypes of ‘generalist’ nematode helminths, which, when common grazing occurs, can be transmitted to sheep and goats [24].

### 4.3. Bacterial Infections

In cases of abortion caused by *Chlamydia abortus* [25] or *Coxiella burnetii* [26], which are frequent aetiological agents of the problem in small ruminant farms in Greece [27,28], shedding of the organisms takes place through the genital excretions and/or the faeces of the infected animals. Specifically in the genital excretions, the organisms can be found when the animals are in oestrus and vaginal excretions abound. Evidence of infection of cervids by *Chlamydia* spp. has been reported in Italy [29] and in Spain [30,31], whilst in Italy cervid infections by *C. burnetii* have also been reported [32]. Moreover, Gonzalez-Barrio et al. [33] have reported the shedding of *C. burnetii* in vaginal excretions by infected red deer, similarly to respective infections in small ruminants. Therefore, common grazing of sheep and goats with cervids may result in increased burden of pastures with abortifacient agents, which can, in turn, result in increased risk of infections for all species. Hence, it may be postulated that cross-infection can occur between sheep/goats and wildlife ruminants, and this is reflected in the findings of the present study. Moreover, vaccination can protect immunised animals, even in front of this higher risk of infection, as a higher incidence of cases of abortion was evident in unvaccinated farms.

The transmission of *Escherichia coli*, the most frequent causal agent of diarrhoea syndrome in newborn lambs/kids, from deer to sheep has been reported to occur through faecal contamination of shared pastures and foraging areas [34,35,36]. In Scotland, where both sheep and deer are significant components of agricultural systems, a strong association was reported for the detection of antibiotic-resistant *E. coli* strains in faecal samples from deer in areas with a concurrent high density of sheep [37]. Further, simultaneous isolation of *Salmonella* spp., another causal agent of diarrhoea syndrome, from deer and sheep grazing on the same pastures has also been reported [38]. Finally, the transmission of less frequent aetiological agents of diarrhoea syndrome has also been suggested; for example, closely related *Rotavirus* strains have been detected in samples from livestock and wildlife ruminants, which supports a hypothesis of occurrence of interspecies transmission [39].

In contrast, however, common grazing with wildlife ruminants was not found to be associated with an increased incidence of cases of lameness in sheep and goats. *Dichelobacter nodosus*, the primary causal agent of foot-rot, has been reported to be a rare pathogen in deer [40,41]. Therefore, the possibility for spreading the pathogen by those species in the grazing fields is small, and that way wildlife ruminants do not contribute to the infection of sheep and goats during grazing.

### 4.4. Predictors

The results of the multivariable analysis revealed that common grazing of sheep and goats with wildlife ruminants was not as significant as management practices and climate variables for the health-related outcomes assessed. Nevertheless, it is noted that the findings have indicated a potential for interactions between livestock and wildlife ruminants, which needs to be taken into account in the development of health management protocols for small ruminant farms. The results of the multivariable analyses indicate that common grazing of sheep/goats with wildlife ruminants overlaps with management practices and climate variables. Thus, the potential impact of livestock—wildlife contact cannot be distinguished from differences in management or in climatological parameters. Possibly, common grazing is a parameter related to management- and climate-related variables rather than a significant independent predictor.

Moreover, one may argue that prospective changes in the environment of farms can support an increased importance of the present findings in the future. For example, the increasing urbanisation may contribute to bringing closer livestock with wildlife or, alternatively, changes in climate variables at farm locations may shift the balance in the relative importance of the various predictors.

In any case, it should be noted that transmission of gastrointestinal pathogens between sheep/goats and wildlife ruminants may occur in both directions, i.e., from wildlife ruminants to livestock and from sheep/goats to deer. This may be a means for the dissemination of pathogens across small ruminant farms, which are in significant distances between them, given that wildlife ruminants can command large areas of habitat and grazing.

## 5. Conclusions

The findings have revealed associations of common grazing with wildlife ruminants with the health of sheep and goats. The current findings provide epidemiological evidence, whilst confirmation of identity similarity of pathogens from livestock and wild mammals has not been attempted.

Nevertheless, the findings indicate implications in the health management of farms, for example, in the administration of anthelmintic treatments and in the development of vaccination programmes in livestock farms. Therefore, it is important to consider wildlife species when addressing the health management in sheep and goat farms.

## Figures and Tables

**Table 1:** Results of parasitological examinations in faecal samples from 369 small ruminant farms in Greece, in accordance with the occurrence of common grazing with wildlife ruminants of the livestock on the farms.

**Common Grazing**	**Frequency (Proportion) of Farms in Which Presence of Parasitic Elements of the Following Helminths Was Detected**																				
	**Dd ^1^**	**Fh ^1^**	**Pc ^1^**	**Mon ^1^**	**Trich** **Fam ^1^**	**Tel ^1^**		**Hc ^1^**		**Trich ^1^**	**Chab ^1^**	**Coop ^1^**		**Buno ^1^**	**Nem ^1^**		**Sp ^1^**	**Trichur ^1^**		**Lung ^1^**	**epg ^2^** **>300**
Yes (*n* = 36)	5 (13.9%)	0 (0.0%)	1 (2.8%)	6 (16.7%)	36 (100.0%)	36 (100.0%)		36 (100.0%)		34 (94.4%)	27 (75.0%)	16 (44.4%)		5 (13.9%)	9 (25.0%)		2 (5.6%)	9 (25.0%)		7 (19.4%)	13 (36.1%)
No (*n* = 333)	56 (16.8%)	1 (0.3%)	4 (1.2%)	72 (21.6%)	308 (92.9%)	308 (92.5%)		307 (92.2%)		286 (85.9%)	230 (69.1%)	154 (46.2%)		85 (25.5%)	64 (19.2%)		22 (6.6%)	71 (21.3%)		70 (21.0%)	77 (23.1%)
*p*-value	0.65	0.74	0.44	0.49	0.09	0.09		0.10		0.15	0.46	0.84		0.12	0.41		0.81	0.61		0.82	0.08
**Common Grazing**	**Median (Interquartile Range) of Respective Parameter in Faecal Samples**																				
	**epg**		**Proportion (%) Tel ^1^**				**Proportion (%) Hc ^1^**		**Proportion (%) Trich ^1^**				**Proportion (%) Chab ^1^**			**Proportion (%) Coop ^1^**			**Proportion (%) Buno ^1^**		
Yes (*n* = 36)	270 (400)		60 (15)				34 (16)		3 (2)				1 (0)			0 (1)			0 (0)		
No (*n* = 333)	150 (225)		62 (14)				31 (16)		2 (2)				1 (1)			0 (1)			0 (1)		
*p*-value	0.06		0.67				0.37		0.55				0.53			0.94			0.12		

^1^ Dd = *Dicrocoelium dendriticum*, Fh = *Fasciola hepatica*, Pc = *Paramphistomum cervi*, Mon = *Moniezia* spp., Tric fam = Trichostrongylidae, Tel = *Teladorsagia* spp., Hc = *Haemonchus contortus*, Trich = *Trichostrongylus* spp., Chab = *Chabertia* spp., Coop = *Cooperia* spp., Buno = *Bunostomum* spp., Nem = *Nematodirus* spp., Sp = *Strongyloides papillosus*, Trichur = *Trichuris* spp., Lung = lungworms ^2^ Trichostrongulidae eggs per gram.

**Table 2:** Median annual incidence (interquartile range) of cases of abortion among 444 small ruminant farms, in accordance with the occurrence of common grazing with wildlife ruminants.

**Incidence of Cases of Abortion in Accordance with Livestock Species on Farms**			
Livestock Species	Common Grazing with Wildlife Ruminants		
	Yes (*n* = 41)	No (*n* = 403)	Yes (*n* = 41)
Sheep (*n* = 325)	1.6% (3.3%)	0.0% (2.6%)	0.05
Goats (*n* = 119)	0.0% (10.3%)	0.0% (3.3%)	0.29
**Incidence of Cases of Abortion in Accordance with Anti-Chlamydial Vaccination** **of Livestock on Farms**			
Vaccination Against *Chlamydia psittaci*	Common Grazing with Wildlife Ruminants		
	Yes (*n* = 41)	No (*n* = 403)	Yes (*n* = 41)
No (*n* = 275)	2.1% (4.2%)	0.0% (2.9%)	0.046
Yes (*n* = 169)	1.2% (7.6%)	0.0% (2.9%)	0.25

**Table 3:** Median (interquartile range) annual incidence of lamb/kid diarrhoea cases, in accordance with the occurrence of common grazing with wildlife ruminants and the livestock species on farms.

Livestock Species	Common Grazing with Wildlife Ruminants		
	Yes (*n* = 41)	No (*n* = 403)	Yes (*n* = 41)
Sheep (*n* = 325)	8.2% (16.8%)	1.7% (9.0%)	0.0008
Goats (*n* = 119)	13.9% (10.9%)	1.8% (12.8%)	0.048

## Data Availability

Data are available upon request from the corresponding author.

## Supplementary Materials

The following supporting information can be downloaded at: https://www.mdpi.com/article/10.3390/ani15243579/s1, Table S1: Published papers derived from Ph.D. thesis cited in the reference list of the main text, to which readers may refer for specific information regarding the citations in the text; Figure S1: Location of the 444 small ruminant farms (locations indicated by red dots) throughout Greece, which were visited during the investigation; Table S2: Questionnaire (with 442 questions) employed in the study for collection of information in sheep or goat farms; Table S3: Information (related to infrastructure, animals, health management, health problems and climatic conditions) obtained by means of a structured questionnaire in 444 small ruminant farms; Table S4: Details of multivariable models (*n* = 2) employed for the evaluation of associations with the common grazing of livestock (sheep/goats) on farms with wildlife ruminants in 325 sheep flocks and 119 goat herds in Greece; Table S5: Climate variables at locations of sheep/goat farms, where common grazing of sheep/goats and wildlife ruminants occurred or did not occur; Table S6: Associations between health-related outcomes and grazing-related variables in sheep/goat farms, with common grazing of sheep/goats and wildlife ruminants; Table S7: Results of the multivariable analysis for faecal counts >300 epg in 369 small ruminant farms in a countrywide investigation in Greece; Table S8: Results of the multivariable analysis for annual incidence of cases of abortion in 444 small ruminant farms in a countrywide investigation in Greece; Table S9: Results of the multivariable analysis for the presence of cases of diarrhoea in lambs/kids in 444 small ruminant farms in a countrywide investigation in Greece; Table S10: Results of the multivariable analysis for annual incidence of cases of diarrhoea in lambs/kids in 444 small ruminant farms in a countrywide investigation in Greece. Refs. [42,43,44,45,46] are cited in Supplementary Materials.
