# Supplementary material for: A Note on Some Health-Related Outcomes in Small Ruminant Farms with Common Grazing with Wildlife Ruminants
Source: Animals (Basel). 2025 Dec 12;15(24):3579. doi: 10.3390/ani15243579 (PMC12729670; doi:10.3390/ani15243579)
Supplement: Supplementary file 1 [file animals-15-03579-s001.zip › animals-4037964-supplementary.pdf]

A Note on Some Health-Related Outcomes in Small Ruminant Farms with Common Grazing with Wildlife Ruminants

E.I. Katsarou, C.K. Michael, K.V. Arsenopoulos, D.T. Lianou, D.V. Liagka, V.S. Mavrogianni, E. Papadopoulos and G.C. Fthenakis

Table S1. Published papers derived from PhD thesis cited in the reference list of the main text, to which readers may refer for specific information regarding the citations in the text.

Lianou, D.T. *Mapping the Small Ruminant Industry in Greece: Health Management and Diseases of Animals, Preventive Veterinary Medicine and Therapeutics, Reproductive Performance, Production Outcomes, Veterinary Public Health, Socio-demographic Characteristics of the Farmers*. PhD thesis, University of Thessaly, Volos, Greece, 2023.	Lianou, D.T.; Arsenopoulos, K.V.; Michael, C.K.; Papadopoulos, E., Fthenakis, G.C. Dairy goats helminthosis and its potential predictors in Greece: findings from an extensive countrywide study. *Vet. Parasitol.* **2023**, *320*, 109962.<br>Lianou, D.T., Arsenopoulos, K.V., Michael, C.K., Papadopoulos, E., Fthenakis, G.C. Helminth infections in dairy sheep found in an extensive countrywide study in Greece and potential predictors for their presence in faecal samples. *Microorganisms* **2023**, *11*, 571.<br>Lianou, D.T., Chatziprodromidou, I.P., Vasileiou, N.G.C., Michael, C.K., Mavrogianni, V.S., Politis, A.P., Kordalis, N.G., Billinis, C., Giannakopoulos, A., Papadopoulos, E., Giannenas, I., Ioannidi, K.S., Katsafadou, A.I., Gougoulis, D.A., Lacasta, D., Caroprese, M., Fthenakis, G.C. A detailed questionnaire for the evaluation of health management in dairy sheep and goats. *Animals* **2020**, *10*, 1489.<br>Lianou, D.T., Michael, C.K., Fthenakis, G.C. Data on mapping 444 dairy small ruminant farms during a countrywide investigation performed in Greece. *Animals* **2023**, *13*, 2044.<br>Lianou, D.T., Michael, C.K., Petinaki, E., Mavrogianni, V.S., Fthenakis, G.C. Administration of vaccines in dairy sheep and goat farms: patterns of vaccination, associations with health and production parameters, predictors. *Vaccines* **2022**, *10*, 1372.

Figure S1. Location of the 444 small ruminant farms (locations indicated by red dots) throughout Greece, which were visited during the investigation.

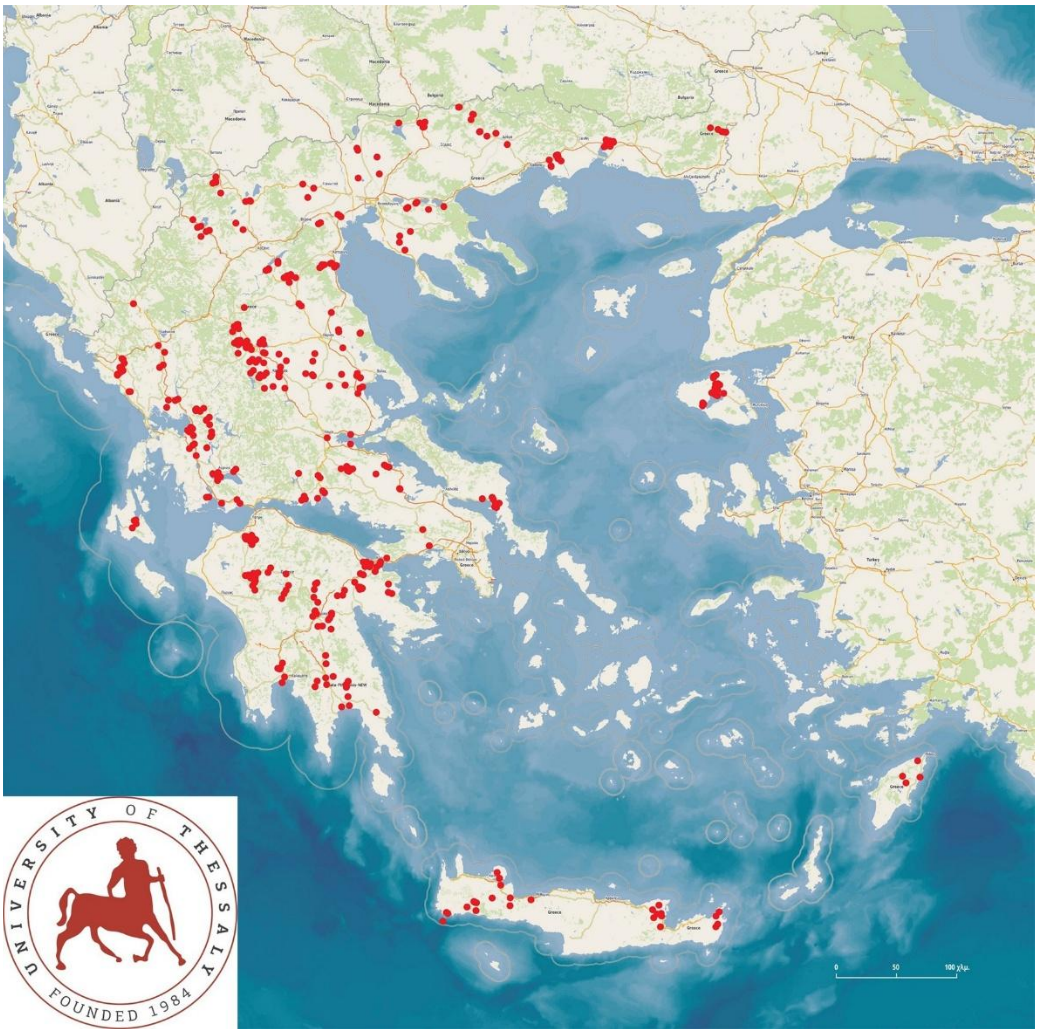

Table S2. Questionnaire (with 442 questions) employed in the study for collection of information in sheep or goat farms.

Availability of questionnaire used in the interviews [Lianou et al. 2020]
https://www.mdpi.com/2076-2615/10/9/1489/s1

Reference.
Lianou, D.T. et al. (2020). A detailed questionnaire for the evaluation of health management in dairy sheep and goats. *Animals*, **2020**, *10*, 1489.

Table S3. Information (related to infrastructure, animals, health management, health problems and climatic conditions) obtained by means of a structured questionnaire in 444 small ruminant farms.

Variables related to infrastructure in farms
Management system applied in farm (EFSA classification: shepherding / intensive / semi-intensive / semi-extensive / extensive / very extensive / mixed)
Altitude at the location of farm (m)
Variables related to animals in farms
No. of ewes / does on farms (no.)
No. of adult sheep / goats on farms (no.)
Total number of lambs / kids born during the preceding lambing season (no.)
Variables related to health management in farms
Common grazing of sheep with wildlife ruminants (yes / no)
Species of wildlife mammals identified in common grazing (description)
Total grazing land by the farm animals (hectares)
Duration of grazing annually (no. of months)
Maximum distance from farm of area grazed (km)
Vaccination against brucellosis (yes / no)
Vaccination against chlamydia infections (yes / no)
Vaccination against *Toxoplasma gondii* abortion (yes / no)
Annual frequency of systemic disinfections in the farm (no. of occasions)
Health problems
Diseases of adult animals – abortion; total cases during the preceding season (no.)
Diseases of adult animals – lameness; total cases during the preceding season (no.)
Diseases of young animals – diarrhoea; total cases during the preceding season (no.)
Variables related to climatic conditions at the locations of farms
Average annual temperature at 2 m (°C)
Average annual temperature of Earth skin (°C)
Average annual minimum temperature at 2 m (°C)
Average annual maximum temperature at 2 m (°C)
Average annual temperature range at 2 m (°C)
Average annual relative humidity at 2 m (%)
Average annual precipitation (kg m^{-2} s^{-1})
Average annual wind speed at 10 m (m s^{-1})

Table S4. Details of multivariable models ($n = 2$) employed for the evaluation of associations with the common grazing of livestock (sheep / goats) on farms with wildlife ruminants in 325 sheep flocks and 119 goat herds in Greece.

Health-related variable	Control to case ratio	Case to variable ratio	Variables offered to the multivariable model	Variables required in the final model
faecal counts > 300 epg	333 / 36	36 / 4	(a) Common grazing of live-stock (sheep / goats) on farms with wildlife ruminants, (b) Month into the lactation period at sampling, (c) Application of reproductive control practices in the farm, (d) Age of lamb / kid removal from their dams	(a) Common grazing of livestock (sheep / goats) on farms with wildlife ruminants, (b) Month into the lactation period at sampling, (c) Application of reproductive control prac-tices in the farm
presence of cases of abortion	403 / 41	41 / 2	(a) Common grazing of livestock (sheep / goats) on farms with wildlife ruminants, (b) Annual precipitation at farm location	(a) Common grazing of livestock (sheep / goats) on farms with wildlife ruminants, (b) Annual precipitation at farm location
annual incidence of cases of abortion	403 / 41	41 / 2	(a) Common grazing of livestock (sheep / goats) on farms with wildlife ruminants, (b) Annual precipitation at farm location	(a) Common grazing of livestock (sheep / goats) on farms with wildlife ruminants, (b) Annual precipitation at farm location
presence of cases of diarrhoea in lambs / kids	403 / 41	41 / 2	(a) Common grazing of livestock (sheep / goats) on farms with wildlife ruminants, (b) Annual temperature range at farm location	(a) Common grazing of livestock (sheep / goats) on farms with wildlife ruminants, (b) Annual temperature range at farm location
annual incidence of cases of diarrhoea in lambs / kids	403 / 41	41 / 2	(a) Common grazing of livestock (sheep / goats) on farms with wildlife ruminants, (b) Annual temperature range at farm location	(a) Common grazing of livestock (sheep / goats) on farms with wildlife ruminants, (b) Annual temperature range at farm location

Table S5. Climate variables at locations of sheep / goat farms, where common grazing of sheep / goats and wildlife ruminants occurred or did not occur.

Variables Related to Climatic Conditions at the Locations of Farms	Locations of Farms, where Common Grazing of Sheep / Goats and Wildlife Ruminants Occurred	Locations of Farms, where Common Grazing of Sheep / Goats and Wildlife Ruminants Did not Occur	*p* Value
Average annual temperature at 2 m (°C)	15.8 (1.3)	16.0 (1.8)	0.0004
Average annual temperature of Earth skin (°C)	15.8 (1.1)	16.3 (2.0)	0.0005
Average annual minimum temperature at 2 m (°C)	−7.6 (3.8)	−5.3 (4.9)	0.033
Average annual maximum temperature at 2 m (°C)	38.0 (1.8)	38.0 (3.6)	0.84
Average annual temperature range at 2 m (°C)	46.9 (5.0)	42.6 (7.5)	0.049
Average annual relative humidity at 2 m (%)	68.4 (2.1)	68.1 (3.9)	0.027
Average annual precipitation (kg m^{-2} s^{-1})	2.2 (0.7)	1.9 (0.7)	0.004
Average annual wind speed at 10 m (m s^{-1})	1.8 (1.0)	2.2 (1.0)	0.012

Table S6. Associations between health-related outcomes and grazing-related variables in sheep / goat farms, with common grazing of sheep / goats and wildlife ruminants.

Grazing-related Variables	Farms with Faecal Counts > 300 epg	Farms with Faecal Counts ≤ 300 epg	*p* Value
Total grazing area by livestock (hectares)	25 (90)	50 (80)	0.36
Maximum distance grazed from the farm (km)	1000 (1300)	1000 (1800)	0.64
Duration of grazing during the year (months)	6 (7)	8 (4)	0.11
**Grazing-related Variables**	**Farms with Presence of Cases of Abortion**	**Farms with no Presence of Cases of Abortion**	***p* Value**
Total grazing area by livestock (hectares)	50 (85)	50 (52.5)	0.55
Maximum distance grazed from the farm (km)	1000 (1900)	1000 (2200)	0.71
Duration of grazing during the year (months)	8 (6)	9 (6)	0.48
**Grazing-related Variables**	**Annual Incidence of Cases of Abortion**		***p* Value**
Total grazing area by livestock (hectares)	$r_{sp} = 0.281$		0.08
Maximum distance grazed from the farm (km)	$r_{sp} = 0.151$		0.35
Duration of grazing during the year (months)	$r_{sp} = -0.134$		0.40
**Grazing-related Variables**	**Farms with Presence of Cases of Diarrhoea in Lambs / Kids**	**Farms with no Presence of Cases of Diarrhoea in Lambs / Kids**	***p* Value**
Total grazing area by livestock (hectares)	50 (90)	50 (57.5)	0.33
Maximum distance grazed from the farm (km)	1000 (1850)	1250 (1500)	0.53
Duration of grazing during the year (months)	8 (6)	12 (4.5)	0.19
**Grazing-related Variables**	**Annual Incidence of Cases of Diarrhoea in Lambs / Kids**		***p* Value**
Total grazing area by livestock (hectares)	$r_{sp} = 0.191$		0.23
Maximum distance grazed from the farm (km)	$r_{sp} = 0.015$		0.92
Duration of grazing during the year (months)	$r_{sp} = -0.128$		0.43

Table S7. Results of the multivariable analysis for faecal counts > 300 epg in 369 small ruminant farms in a countrywide investigation in Greece.

Variables	Odds Ratio [1] (95% Confidence Intervals)	*p* Value
Month into the lactation period at sampling		0.003
0–1 (0/9: 0.0%)	reference	--
2–5 (43/203: 21.2%)	5.150 (0.294-90.237)	0.26
6–9 (39/141: 27.7%)	7.322 (0.416–128.810)	0.17
10 and thereafter (8/16: 50.0%)	19.000 (0.947–381.084)	0.05
Application of reproductive control practices on the farm		0.047
Yes (15/86 = 17.4%)	reference	--
No (75/283 = 26.5%)	1.707 (0.922–3.161)	0.09

[1] odds ratio calculated against the lowest prevalence associations of the variable.

Table S8. Results of the multivariable analysis for annual incidence of cases of abortion in 444 small ruminant farms in a countrywide investigation in Greece.

Variables	Relative Risk (± standard error)	*p* Value
Average annual precipitation		0.006
Per unit of precipitation (kg m^{-2} s^{-1}) increase	1.032 ± 1.011	0.004

Table S9. Results of the multivariable analysis for the presence of cases of diarrhoea in lambs / kids in 444 small ruminant farms in a countrywide investigation in Greece.

Variables	Odds Ratio [1] (95% Confidence Intervals) / Relative Risk (± standard error)	*p* Value
Common grazing of livestock (sheep / goats) with wildlife ruminants		0.001
Yes (35/41 = 85.4%)	4.477 (1.842 – 10.883)	0.0009
No (228/403 = 56.6%)	reference	--
Average annual temperature range		0.002
Per unit of temperature (°C) increase	1.010 ± 1.003	0.003

[1] odds ratio calculated against the lowest prevalence associations of the variable.

Table S10. Results of the multivariable analysis for annual incidence of cases of diarrhoea in lambs / kids in 444 small ruminant farms in a countrywide investigation in Greece.

Variables	Relative Risk (± standard error)	*p* Value
Average annual temperature range		0.0004
Per unit of temperature (°C) increase	1.004 ± 1.001	0.0002
